# Supplementary material for: A Chimeric IL-15/IL-15Rα Molecule Expressed on NFκB-Activated Dendritic Cells Supports Their Capability to Activate Natural Killer Cells
Source: Int J Mol Sci. 2021 Sep 23;22(19):10227. doi: 10.3390/ijms221910227 (PMC8508776; doi:10.3390/ijms221910227)
Supplement: Supplementary file 1 [file ijms-22-10227-s001.zip › ijms-1342074-supplementary.pdf]

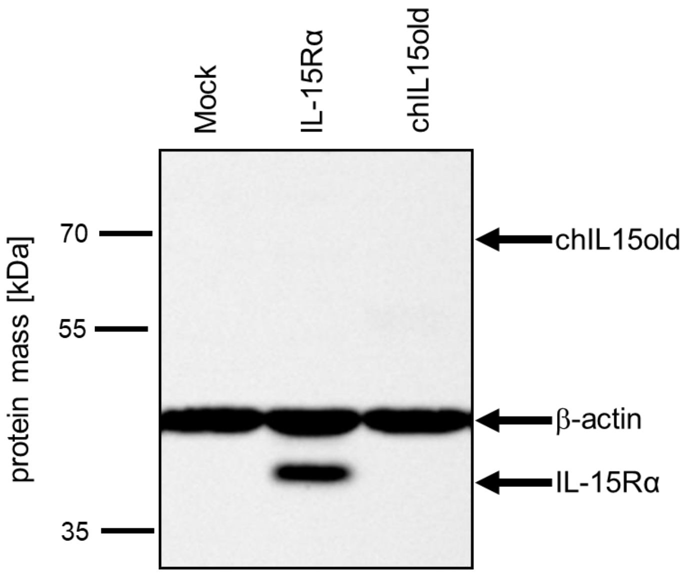

Supplementary Figure S1: No detection of chIL15old by Western blot

DCs were generated from monocytes and matured with the standard maturation cocktail. These cmDCs were electroporated without RNA (Mock) or with RNA coding for wild type IL-15R*α* or chIL15old. Cell pellets were frozen 4 h after electroporation (EP). To analyze the expression of the IL-15R*α* and Super IL-15, Western blotting experiments were performed with an anti-human IL-15R*α* antibody (clone JM7A4). Anti-β-actin-staining served as loading control. The theoretical size of IL-15R*α* (40 kDa) and chIL15old (70 kDa) are indicated. The depicted blot is representative for 3 independent experiments.

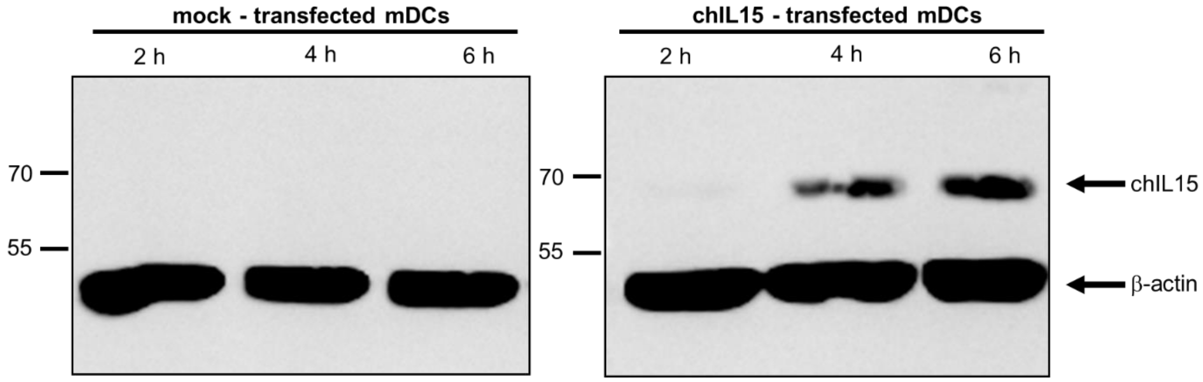

Supplementary Figure S2: Detection of chIL15 by Western blot

DCs were generated from monocytes and matured with the standard maturation cocktail. These cmDCs were electroporated without RNA (Mock) or with RNA coding for chIL15. Cell pellets were frozen 2, 4, and 6 h after electroporation. To analyze the expression of CD25-SIL-15, Western blotting experiments were performed with an anti-human IL-15R*α* antibody (clone JM7A4). Anti-β-actin-staining served as loading control. The theoretical size of chIL15 (70 kDa) is indicated. The depicted blots are one representative out of 3 independent experiments.

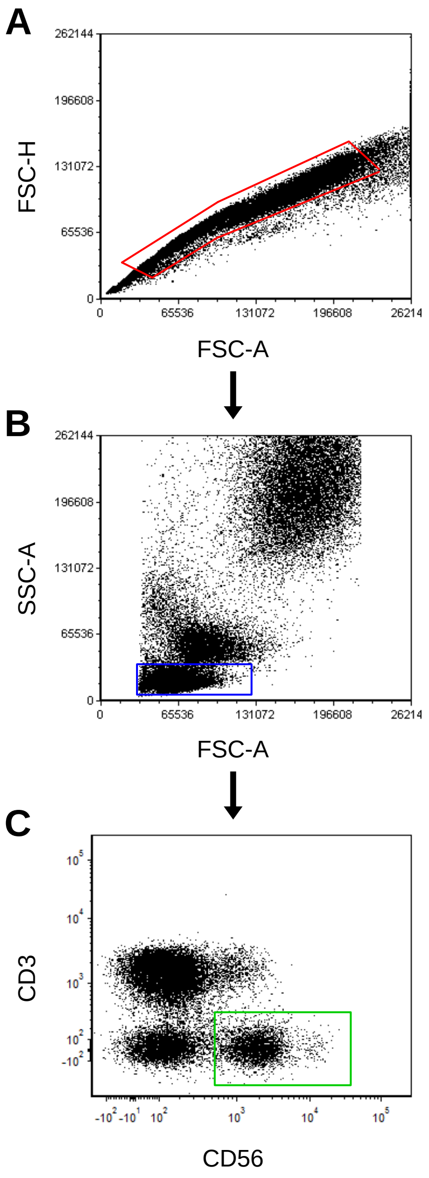

Supplemental Figure S3: Gating strategy for NK cell identification via flow cytometry.

(**A**) First, DC and PBMC co-cultures were gated on live and dead cells (red gate). (**B**) Lymphocytes were detected *via* forward and side-scatter (blue gate). (**C**) Through staining with antibodies specific for CD56 and CD3, $CD56^{+}/CD3^{-}$ NK cells were detected.

Supplementary Data file S4: Sequence of mRNA encoding the chimeric proteins chIL15old and chIL15.

Color coding:

Light gray: IL15 signal peptide

Dark gray: IL15 premature sequence

Green: CD25 signal peptide

Magenta: mature IL15

Unmarked: Spacer

Cyan: IL15Rα

chIL15old

atgcggatcagcaagccccacctgagatccatcagcatccagtgctacctgtgcctgctg

M R I S K P H L R S I S I Q C Y L C L L

ctgaacagccacttcctgaccgaggccggcatccacgtgttcatcctgggctgcttttct

L N S H F L T E A G I H V F I L G C F S

gccggcctgcccaagacagaggccaattgggttaacgttatcagcgatttaaagaagatc

A G L P K T E A N W V N V I S D L K K I

gaagatctgatccagtccatgcatatcgacgccaccctgtacaccgagagcgacgtgcac

E D L I Q S M H I D A T L Y T E S D V H

cccagctgcaaagtgaccgccatgaagtgctttctgctggaactgcaagtgatcagcctg

P S C K V T A M K C F L L E L Q V I S L

gaaagcggcgacgccagcatccacgacaccgtggaaaacctgatcatcctggccaacaac

E S G D A S I H D T V E N L I I L A N N

agcctgagcagcaacggcaacgtgaccgagtctggctgcaaagagtgcgaggaactggaa

S L S S N G N V T E S G C K E C E E L E

gagaagaatatcaaagagttcctgcagagcttcgtgcacatcgtgcagatgttcatcaac

E K N I K E F L Q S F V H I V Q M F I N

accagcagcggcggaggctctggcggaggcggatctgggggcggaggatcaggcgggga

T S S G G G S G G G G S G G G G S G G G

ggaagtgggggaggctctctgcagatcacctgtcccccacccatgagcgtggaacacgcc

G S G G G S L Q I T C P P P M S V E H A

gacatctgggtcaagagctacagcctgtacagccgggaacggtacatctgcaacagcggc

D I W V K S Y S L Y S R E R Y I C N S G

ttcaagcggaaggccggcacaagcagcctgacagagtgcgtgctgaacaaggccaccaac

F K R K A G T S S L T E C V L N K A T N

gtggcccactggaccacccctagcctgaagtgcatcagagatcccgccctggtgcatcag

V A H W T T P S L K C I R D P A L V H Q

cggccagcccctccaagcacagtgacaacagctggcgtgacccccccagcctgagagcctg

R P A P P S T V T T A G V T P Q P E S L

agcccttctggaaaagagcctgccgccagcagccccagcagcaacaatactgccgccacc

S P S G K E P A A S S P S S N N T A A T

acagccgccatcgtgcctggctctcagctgatgcctagcaagagccctagcaccggcacc

T A A I V P G S Q L M P S K S P S T G T

accgagatcagcagccacgagtctagccacggcaccccatctcagaccaccgccaagaac

T E I S S H E S S H G T P S Q T T A K N

tgggagctgacagccagcgcctctcaccagcctccaggcgtgtaccctcagggccacagc

W E L T A S A S H Q P P G V Y P Q G H S

gataccacagtggccatcagcacctccaccgtgctgctgtgtggactgagcgccgtgtct

D T T V A I S T S T V L L C G L S A V S

ctgctggcctgctacctgaagtccagacagacccctccactggccagcgtggaaatggaa

L L A C Y L K S R Q T P P L A S V E M E

gccatggaggccctgcctgtgacctggggcaccagctccagagatgaggacctcgagaac

A M E A L P V T W G T S S R D E D L E N

tgcagccaccatctctga

C S H H L -

chIL15

atggattcatacctgctgatgtggggactgctcacgttcatcatggtgcctggctgccag

M D S Y L L M W G L L T F I M V P G C Q

gccaattgggttaacgttatcagcgatttaaagaagatcgaagatctgatccagtccatg

A N W V N V I S D L K K I E D L I Q S M

catatcgacgccaccctgtacaccgagagcgacgtgcaccccagctgcaaagtgaccgcc

H I D A T L Y T E S D V H P S C K V T A

atgaagtgctttctgctggaactgcaagtgatcagcctggaaagcggcgacgccagcatc

M K C F L L E L Q V I S L E S G D A S I

cacgacaccgtggaaaacctgatcatcctggccaacaacagcctgagcagcaacggcaac

H D T V E N L I I L A N N S L S S N G N

gtgaccgagtctggctgcaaagagtgcgaggaactggaagagaagaatatcaaagagttc

V T E S G C K E C E E L E E K N I K E F

ctgcagagcttcgtgcacatcgtgcagatgttcatcaacaccagcagcggcggaggctct

L Q S F V H I V Q M F I N T S S G G G S

ggcggaggcggatctgggggcggaggatcaggcgggggaggaagtgggggaggctctctg

G G G G S G G G G S G G G G S G G G S L

cagatcacctgtcccccacccatgagcgtggaacacgccgacatctgggtcaagagctac

Q I T C P P P M S V E H A D I W V K S Y

agcctgtacagccgggaacggtacatctgcaacagcggcttcaagcggaaggccggcaca

S L Y S R E R Y I C N S G F K R K A G T

agcagcctgacagagtgcgtgctgaacaaggccaccaacgtggcccactggaccacccct

S S L T E C V L N K A T N V A H W T T P

agcctgaagtgcatcagagatcccgccctggtgcatcagcggccagcccctccaagcaca

S L K C I R D P A L V H Q R P A P P S T

gtgacaacagctggcgtgacccccccagcctgagagcctgagcccttctggaaaagagcct

V T T A G V T P Q P E S L S P S G K E P

gccgccagcagccccagcagcaacaatactgccgccaccacagccgccatcgtgcctggc

A A S S P S S N N T A A T T A A I V P G

tctcagctgatgcctagcaagagccctagcaccggcaccaccgagatcagcagccacgag

S Q L M P S K S P S T G T T E I S S H E

tctagccacggcaccccatctcagaccaccgccaagaactgggagctgacagccagcgcc

S S H G T P S Q T T A K N W E L T A S A

tctcaccagcctccaggcgtgtaccctcagggccacagcgataccacagtggccatcagc

S H Q P P G V Y P Q G H S D T T V A I S

acctccaccgtgctgctgtgtggactgagcgccgtgtctctgctggcctgctacctgaag

T S T V L L C G L S A V S L L A C Y L K

tccagacagacccctccactggccagcgtggaaatggaagccatggaggccctgcctgtg

S R Q T P P L A S V E M E A M E A L P V

acctggggcaccagctccagagatgaggacctcgagaactgcagccaccatctctga

T W G T S S R D E D L E N C S H H L -
